# Spatiotemporal-based clusters as a method for dengue surveillance

**DOI:** 10.26633/RPSP.2017.162

**Published:** 2017-12-12

**Authors:** Mayara Romero Canal, Elis Regina da Silva Ferreira, Cássia Fernanda Estofolete, Andréia Martiniano Dias, Caroline Tukasan, Ana Carolina Bertoque, Vitor Dantas Muniz, Maurício Lacerda Nogueira, Natal Santos da Silva

**Affiliations:** 1 Faculdade de Medicina União das Faculdadesn dos Grandes Lagos São José do Rio Preto São Paulo Brasil Faculdade de Medicina, União das Faculdadesn dos Grandes Lagos, São José do Rio Preto, São Paulo, Brasil.; 2 Laboratório de Pesquisas em Virologia Faculdade de Medicina de São José do Rio Preto São Paulo Brasil Laboratório de Pesquisas em Virologia, Faculdade de Medicina de São José do Rio Preto, São Paulo, Brasil.; 3 Laboratório de Modelagens Matemática e Estatística em Medicina União das Faculdades dos Grandes Lagos, São José do Rio Preto São Paulo Brasil Laboratório de Modelagens Matemática e Estatística em Medicina, União das Faculdades dos Grandes Lagos, São José do Rio Preto, São Paulo, Brasil

**Keywords:** Geographic information systems, dengue, public health surveillance, communicable diseases, emerging, Brazil, Sistemas de información geográfica, dengue, vigilancia en salud pública, enfermedades transmisibles emergentes, Brasil, Sistemas de informação geográfica, dengue, vigilância em saúde pública, doenças transmissíveis emergentes, Brasil

## Abstract

**Objectives.:**

*To develop and demonstrate the use of a new method for epidemiological surveillance of dengue*.

**Methods.:**

*This was a retrospective cohort study using data from the Health Department of São José do Rio Preto (São Paulo, Brazil). The geographical coordinates were obtained using QGIS™ (Creative Commons Corporation, Mountain View, California, United States), based on patient addresses in the dengue notification system of the Government of Brazil. SaTScan™ (Martin Kulldorff, Boston, Massachusetts, United States) was then used to create a space-time scan analysis to find statistically significant clusters of dengue. These results were plotted and visualized using Google Earth™ mapping service (Google Incorporated, Mountain View, California, United States)*.

**Results.:**

*More clusters were detected when the maximum number of households per cluster was set to 10% (11 statistically significant clusters) rather than 50% (8 statistically significant clusters). The cluster radius varied from 0.18 – 2.04 km and the period of time varied from 6 days – 6 months. The infection rate was more than 0.5 cases/household*.

**Conclusions.:**

*When using SaTScan for space-time analysis of dengue cases, the maximum number of households per cluster should be set to 10%. This methodology may be useful to optimizing dengue surveillance systems, especially in countries where resources are scarce and government programs have not had much success controlling the disease*.

## RESUMEN

**Objetivos.:**

Elaborar un método nuevo para la vigilancia epidemiológica del dengue y hacer una demostración sobre su uso.

**Métodos.:**

Se realizó un estudio retrospectivo de cohortes usando datos del Departamento de Salud de São José do Rio Preto (São Paulo, Brasil). Partiendo de la dirección de los pacientes en el sistema de notificación del dengue del Gobierno de Brasil, se usó QGIS™ (Creative Commons Corporation, Mountain View, California, Estados Unidos) para obtener sus coordenadas geográficas. Luego se usó SaTScan™ (Martin Kulldorff, Boston, Massachusetts, Estados Unidos) para crear un análisis de exploración espacio-temporal a fin de detectar conglomerados de dengue estadísticamente significativos. Por último, se usó el servicio de mapas de Google Earth™ (Google Incorporated, Mountain View, California, Estados Unidos) para graficar y visualizar estos resultados.

**Resultados.:**

Se detectaron más conglomerados cuando el número máximo de viviendas por conglomerado se estableció en 10% (11 conglomerados estadísticamente significativos) en lugar de 50% (8 conglomerados estadísticamente significativos). El radio de los conglomerados se ubicó entre 0,18 y 2,04 km, y la duración entre 6 días y 6 meses. La tasa de infección fue de más de 0,5 casos por vivienda.

**Conclusiones.:**

Cuando se usa SaTScan, el número máximo de viviendas por conglomerado debe establecerse en 10% para el análisis de exploración. Este método puedeser útil para optimizar los sistemas de vigilancia del dengue, especialmente en aquellos países donde los recursos son escasos y los programas gubernamentales no han tenido mucho éxito para controlar la enfermedad.

## RESUMO

**Objetivos.:**

Desenvolver um novo método de vigilância epidemiológica da dengue e demonstrar a sua aplicação.

**Métodos.:**

Estudo de coorte retrospectiva conduzido com dados obtidos da Secretaria de Saúde de São José do Rio Preto, Estado de São Paulo, Brasil. As coordenadas geográficas foram obtidas com o software QGIS™ (Creative Commons Corporation, Mountain View, Califórnia, EUA) segundo os endereços dos pacientes registrados no sistema de notificação do Programa Nacional de Controle da Dengue do Ministério da Saúde do Brasil. Foi usado o software SaTScan™ (Martin Kulldorff, Boston, Massachusetts, EUA) com o método Scan espaçotemporal para a detecção de conglomerados espaciais de casos de dengue com significância estatística. Os resultados foram representados graficamente e visualizados com a ferramenta de geomapeamento Google Earth™ (Google Incorporated, Mountain View, Califórnia, EUA).

**Resultados.:**

Detectou-se um número maior de conglomerados espaciais ao se estabelecer o número máximo de domicílios por conglomerado em 10% em comparação a 50% (11 versus 8 conglomerados com significância estatística). O raio dos conglomerados espaciais detectados variou de 0,18 km a 2,04 km e o período de tempo oscilou entre 6 dias e 6 meses. A taxa de infecção foi superior a 0,5 caso por domicílio.

**Conclusões.:**

O número máximo de domicílios por conglomerado deve ser de 10% ao conduzir a análise da distribuição espacial com o SaTScan. Este método pode contribuir para melhorar o desempenho dos sistemas de vigilância da dengue, sobretudo nos países com recursos limitados e resultados insatisfatórios nos programas nacionais de controle da doença.

Dengue is a viral disease that affects approximately 50 million – 100 million people annually and puts an estimated 3.6 billion at risk worldwide (1, 2). Brazil alone reported over 1.5 million cases in 2015 (3).

There are four dengue virus serotypes (DENV-1, DENV-2, DENV-3, and DENV-4), which when simultaneously present in a population, create conditions that increase incidence and complicate dengue control efforts (4). Government agencies charged with dengue control and implement face significant challenges—continuously building and supporting teams that educate the population at risk,treat patients,strategies for eliminating the vector and its breeding sites (1). Moreover, dengue control strategies are not always successful because health workers often find it difficult to apply the guidelines set by the government health agencies. This is due to various reasons, such as inaccessible terrain,incorrect patient addresses, and an unwillingness of residents to welcome health workers (1). In this context, it is essential to use established methods, as well as innovative approaches, to combat dengue fever (5, 6).

One innovation with potential is the geoprocessing of dengue data, a process that can improve visualization of the spatial and temporal distribution of events. This information can, in turn, provide a more comprehensive understanding of a situation and can aid in generating new hypotheses and formulating preventive measures (6–9). It can also allow health officials to easily identify priority areas and better direct efforts to control dengue (1, 6, 10, 11). Similar processes have been proposed to combat malaria in the Brazilian Amazon (12), as well as infections in hospitals (13). However, no studies have used a geoprocessing approach in conjunction with free programs that can map dengue cases based solely on the probable address of the infection site. Therefore, the purpose of this study was to develop and demonstrate the use of a new, low-cost, epidemiological method for dengue surveillance based on geoprocessing techniques.

## MATERIALS AND METHODS

This was a retrospective study of confirmed cases of dengue in the Health Department of São José do Rio Preto (São Paulo, Brazil) in 2009. The area has an estimated population of 409 000, with a population density of 945 inhabitants per km2 (14). The study used information on dengue notifications together with free software programs to develop an auxiliary method of surveillance based on patient addresses and to identify priority areas for dengue control teams.

### Data source

Information was collected from patient medical records available in the *Sistema de Informação de Agravos de Notificação* (Information System for Notifiable Diseases; SINAN) for the Health Department of São José do Rio Preto. Every dengue case confirmed in January – December 2009 was evaluated. All dengue cases were confirmed by enzyme-linked immunosorbent assay, non-structural protein 1, or polymerase chain reaction amplification. The notifications that did not contain adequate information about the patient’s address were used as exclusion criterion. Of the total, 72 inappropriate records were excluded and 1 051 cases were submitted to analysis. The year 2009 was chosen because it had the most reliable information available for validating the proposed method. The patient’s address was used as the probable place of infection.

### Geoprocessing of cases and statistical analyses

The address of the primary residence for each dengue patient was entered into QGIS™ version 2.2 (Creative Commons Corporation, Mountain View, California, United States), an opensource geographic information system used to locate geographic coordinates. Then, SaTScan™ version 9.4.2 (Martin Kulldorff, Harvard Medical School and Harvard Pilgrim Health Care Institute, Boston, Massachusetts, United States) was used to analyze the space-time scan. It systematically creates circular windows throughout the geographic area over time. Then it identifies statistically significant clusters of cases within a circle compared to the incidence outside. (15). To identify clusters, the aggregated time length was set at 7 days. The radius of the circular scanning window around each geographical point was defined as the maximum percentage of households to be included in the cluster during the study period. The maximum radius was set at 50% in order to detect large clusters and 10% to detect smaller clusters. The maximum duration of temporal clusters was 50% of the study period.

The location and dimensions of the window associated with the largest likelihood value were used to define the most likely cluster. The significance of this cluster was tested by a probabilistic analysis using the Monte Carlo method (999 replications), as well as a retrospective space-time analysis. Clusters with a *P*-value < 0.05 were considered statistically significant.

SaTScan™ then generated databases of clusters that were plotted for spatial visualization using Google Earth™ mapping service version 7.1.2.2041 (Google Incorporated, Mountain View, California, United States).

### Ethics

This study was authorized by the Ethical Committee from União das Faculdades dos Grande Lagos (São José do Rio Preto, São Paulo, Brazil). It did not include informed consent because patient data remained confidential. Only the researcher involved in the statistical analysis had access to all of the information.

## RESULTS

**FIGURE 1.**
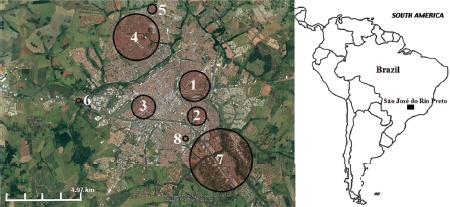
Spatial location of statistically significant clusters of confirmed cases of dengue, with the maximum number of households in the study area set to 50%, in a study of a spatiotemporal method of dengue surveillance, in São José do Rio Preto, São Paulo, Brazil, January – December 2009.

The spatial analysis with the maximum number of households set at 50% identified eight statistically significant clusters within the study period (Figure 1). Two additional clusters were also identified, but these were not statistically significant (*P* = 0.44 and *P* = 0.96, respectively; Table 1). The 1 051 cases of dengue were related to 924 households representing an overall infection rate of 1.14 cases/household. The radii of these statistically significant clusters ranged from 0.18 – 2.04 km. These clusters were scattered around the study area and in different time intervals. A large temporal cluster of 6 months (cluster 4) was detected for May – November 2009. This cluster was also the second largest spatial cluster, with a radius of 1.5 km (Table 2). Infection rates in the clusters were relatively high; three-quarters of them had infection rates above 50%. In particular, cluster 1, which included 86 households, had a very high infection rate(0.86 cases/household). However, cluster 5 had the highest infection rate cases/household), but contained only five households (Table 1).

**FIGURE 2.**
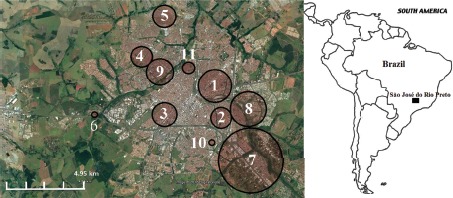
Spatial location of statistically significant clusters of confirmed cases of dengue, with the maximum number of households in the study area set to 10%, in a study of a spatiotemporal method of dengue surveillance in São José do Rio Preto, São Paulo, Brazil, January – December 2009.

When only 10% of households were included in each cluster, the analysis identified 11 statistically significant clusters (*P* < 0.05; Figure 2), in addition to 9 that were not statistically significant (*P* > 0.05; Table 1). The radii of the statistically significant clusters were similar whether the maximum was set at 10% or 50%. Clusters 5 and 8 increased in size, but cluster 4 decreased to less than one-half of its original when the maximum number of households was lowered to 10%. At 10%, cluster 5 had the highest temporal coverage during the study period (Table 2); and the infection rates for 8 of the 11 (72.7%) clusters were statistically significant with values > 0.5 cases/household. In particular, cluster 1 had an infection rate of 0.86 cases/household, with the maximum set at either 10% or 50%. Cluster 11 showed the same infection rate as cluster 1, but included fewer cases of dengue and fewer affected households (Table 1).

***Source:***Prepared by the authors from the study data.

***Source:***Prepared by the authors from the study data.

## DISCUSSION

Although the popularity of geoprocessing to study the dynamics of diseases, especially dengue (16), is increasing, no studies have simultaneously used three open-access programs as an epidemiological surveillance method. In our study, more clusters were detected when the maximum number of households was set to 10% than when it was set to 50% (11 statistically significant clusters versus 8 statistically significant clusters,respectively). In addition, the radii varied (0.18 km – 2.04 km) as did the time duration (6 days – 6 months), and the infection rate was greater than 0.5 cases/household).

The application of such resources for understanding the spatial and temporal distribution of dengue is becoming more popular, and work is being conducted by researchers in several countries (16, 17). For example, in 2014, Banu and colleagues (1) used data from 82 countries in the Asia-Pacific region collected in 1995 – 2004 and a similar methodology to demonstrate the importance of processing dengue data for better prevention and control. However, our approach to identifying clusters using a space-time scan analysis with two scales is more appropriate than a purely spatial scan (1, 11, 18). Similarly, although other units—zip-codes, neighborhoods, cities, states—may be used, geographical coordinates are widely applied as the spatial unit of aggregation in geoprocessing studies of infectious and non-infectious diseases (11). In addition, we set the aggregation time as 7 days to facilitate weekly government surveillance surveys, and in turn, to help guide field teams; however, times as short as a day may also be used (11), especially in areas with a high incidence of dengue.

Furthermore, setting the maximum number of households at 50%, as in our study, has previously been employed and questioned by other authors (19). They agree that this may contribute to the loss of smaller, statistically significant clusters (because they remain undetected). Thus, it is quite reasonable to reduce this percentage to 10%, as in our study. In addition, although any Geographic Information System program can geolocate data points on a map (20), Google Earth™ is more accurate and more informative, and the exact address of cases can be properly georeferenced, including the location, as well as a single view of each cluster household.

**Limitations.** Despite several strengths, this study also has some limitations. For instance, this study was based on retrospective data that was taken from information collected by various health professionals over a year. However, the data were carefully filtered and only data that had no conflicting information was used, i.e., the case of dengue was confirmed and the patient’s primary address was properly recorded.

In addition, the primary residence may not have been the location where the person was bitten by the infected mosquito; however, this may not be problematic since dengue infections are more likely to involve several members of a household (11). Therefore, these limitations may have little influence on the results.

### Conclusions

Our results show that this auxiliary methodology of dengue surveillance may more accurately detect priority areas for intervention when the maximum number of families included in each cluster is adjusted to 10% of total households. This method may be extremely relevant in countries where resources are scarce and government programs have not had much success in controlling dengue. In addition, observing clusters in more detail can lead to the development of community education and awareness programs for improved dengue prevention. However, more studies are needed to better validate the use of spatiotemporal-based clusters as a method for dengue surveillance. We recommend the application of this dengue surveillance methodology in a prospective cohort in future studies.

**TABLE 1.** Characteristics and identification of statistically significant spatiotemporal clusters of confirmed cases of dengue, with the maximum number of households set to 50% and 10%, in a study of a spatiotemporal method of dengue surveillance in São José do Rio Preto, São Paulo, Brazil, January – December 2009

Clusters with 50% of households				Clusters with 10% of households			
Cluster	Dengue cases	Number of households	Infection rate	P	Dengue cases	Number of households	Infection rate	P
1	74	86	0.86	< 0.001	74	86	0.86	< 0.001
2	9	21	0.43	< 0.001	9	21	0.43	< 0.001
3	44	55	0.80	< 0.001	44	55	0.80	< 0.001
4	76	129	0.59	< 0.001	22	38	0.58	< 0.001
5	5	5	1.00	0.001	18	35	0.51	0.001
6	4	5	0.80	0.002	4	5	0.80	0.002
7	9	32	0.28	0.003	9	32	0.28	0.003
8	16	23	0.70	0.03	22	33	0.67	0.01
9	5	7	0.71	0.44	5	19	0.26	0.02
10	13	30	0.43	0.96	16	23	0.70	0.03
11	NAa	NA	NA	NA	12	14	0.86	0.03
12	NA	NA	NA	NA	8	14	0.57	0.16
13	NA	NA	NA	NA	2	2	1.00	0.31
14	NA	NA	NA	NA	5	30	0.17	0.64
15	NA	NA	NA	NA	13	39	0.33	0.71
16	NA	NA	NA	NA	8	12	0.67	0.81
17	NA	NA	NA	NA	24	39	0.62	0.94
18	NA	NA	NA	NA	13	30	0.43	0.96
19	NA	NA	NA	NA	2	2	1.00	0.99
20	NA	NA	NA	NA	3	5	0.60	0.99

**TABLE 2.** The geographical coordinates, radii, and time intervals for statistically significant space-time clusters of confirmed dengue cases identified in the study area, with the maximum number of households set to 50% and 10%, in a study of a spatiotemporal method of dengue surveillance in São José do Rio Preto, São Paulo, Brazil, January – December 2009

Clusters with 50% of households			Clusters with 10% of households		
Cluster	Coordinates	Radius (Km)	Time interval (year/month/day)	Coordinates	Radius (Km)	Time interval (year/month/day)
1	20.801530 S; 49.366388 W	1.02	2009/10/30 – 2009/12/31	20.801530 S; 49.366388 W	1.02	2009/10/30 – 2009/12/31
2	20.819606 S; 49.363143 W	0.63	2009/02/06 – 2009/03/12	20.819606 S; 49.363143 W	0.63	2009/02/06 – 2009/03/12
3	20.816602 S; 49.397446 W	0.77	2009/03/06 – 2009/04/30	20.816602 S; 49.397446 W	0.77	2009/03/06 – 2009/04/30
4	20.775275 S; 49.405045 W	1.50	2009/05/22 – 2009/11/05	20.783507 S; 49.409990 W	0.64	2009/06/05 – 2009/07/23
5	20.758153 S; 49.396602 W	0.30	2009/08/07 – 2009/09/17	20.761840 S; 49.395240 W	0.70	2009/07/24 – 2009/12/03
6	20.815689 S; 49.438960 W	0.18	2009/09/04 – 2009/10/01	20.815689 S; 49.438960 W	0.18	2009/09/04 – 2009/10/01
7	20.843920 S; 49.346334 W	2.04	2009/03/20 – 2009/03/26	20.843920 S; 49.346334 W	2.04	2009/03/20 – 2009/03/26
8	20.833196 S; 49.369322 W	0.18	2009/05/22 – 2009/06/11	20.815154 S; 49.346932 W	1.12	2009/12/04 – 2009/12/31
9	NA^a^	NA	NA	20.792503 S; 49.399189 W	0.86	2009/01/30 – 2009/02/19
10	NA	NA	NA	20.833196 S;49.369322 W	0.18	2009/05/22 – 2009/06/11
11	NA	NA	NA	20.790688 S; 49.381378 W	0.37	2009/05/29 – 2009/06/25

***Source:*** Prepared by authors, based on study results.

a Not applicable.

### Acknowledgements.

The authors wish to thank the Municipal Secretariat for Health of São José do Rio Preto for granting access to the dengue database.

### Funding.

This work was supported by Fundação de Amparo à Pesquisa do Estado de São Paulo (FAPESP-2013/21719-3) to MLN, who is a CNPq Research Fellow.

### Disclaimer.

Authors hold sole responsibility for the views expressed in the manuscript, which may not necessarily reflect the opinion or policy of the RPSP/PAJPH and/or PAHO.
